# A multi-center cross-sectional study on identification of influencing factors of medical students’ emotional engagement in China

**DOI:** 10.1186/s12909-023-04504-w

**Published:** 2023-11-07

**Authors:** Runzhi Huang, Guoyang Zhang, Zhitong Zhou, Min Lin, Shuyuan Xian, Meiqiong Gong, Huabin Yin, Tong Meng, Xin Liu, Xiaonan Wang, Yue Wang, Wenfang Chen, Chongyou Zhang, Erbin Du, Qing Lin, Hongbin Wu, Zongqiang Huang, Jie Zhang, Dayuan Xu, Shizhao Ji

**Affiliations:** 1 Department of Burn Surgery, the First Affiliated Hospital of Naval Medical University, Shanghai, People’s Republic of China; 2 Research Unit of key techniques for treatment of burns and combined burns and trauma injury, Chinese Academy of Medical Sciences, Shanghai, People’s Republic of China; 3 Maastricht University School of Health Professions Education, Maastricht, The Netherlands; 4 Tongji University School of Medicine, Shanghai, 200092 China; 5 Mental Health Education and Consultation Center, Chongqing Medical University, 61 Daxuecheng Middle Road, Chongqing, 401331 China; 6 Office of Educational Administration, Shanghai University, Shanghai, 200444 China; 7 Department of Orthopedics, School of Medicine, Shanghai General Hospital, Shanghai Jiaotong University, 100 Haining Road, Shanghai, China; 8 Department of Rheumatology and Immunology, Second Affiliated Hospital of Naval Medical University, Shanghai, China; 9 Department of Epidemiology and Health Statistics, School of Public Health, Capital Medical University, 10 Xitoutiao, Beijing, 100069 China; 10 Department of Health Statistics, School of Public Health, Air Force Medical University, No.169, Changle West Road, Xi’an, 710032 China; 11 Faculty of Medicine, Jinggangshan University, 28 Xueyuan Road, Ji’An, 343009 China; 12 Basic Medical College, Harbin Medical University, 157 Baojian Road, Harbin, Heilongjiang 150081 China; 13 Frist Clinical Medical College, Mudanjiang Medical University, 66 Tongxiang Street, Mudanjiang, 157011 China; 14 Department of Human Anatomy, Laboratory of Clinical Applied Anatomy, School of Basic Medical Sciences, Fujian Medical University, 1 Xuefu North Road, Fuzhou, 350122 China; 15 National Centre for Health Professions Education Development, Peking University, Beijing, 100191 China; 16 Institute of Medical Education, Peking University, 5 YiHeYuan Road, Beijing, 100871 China; 17 Department of Orthopedics, The First Affiliated Hospital of Zhengzhou University, 1 Jianshe East Road, Zhengzhou, 450052 China; 18 Department of Gynecology, Shanghai First Maternity and Infant Hospital, Tongji University School of Medicine, 2699 Gaoke West Road, Shanghai, 201204 China

**Keywords:** Medical students, Emotional engagement, Influencing factors, Multi-center, Cross-sectional study, Nomogram

## Abstract

**Background:**

Studies exploring influencing factors of emotional engagement among medical students are scarce. Thus, we aimed to identify influencing factors of medical students’ emotional engagement.

**Methods:**

We carried out a multi-center cross-sectional study among 10,901 medical students from 11 universities in China. The Chinese version of Utrecht Work Engagement Scale-Student version (UWES-S) was used to evaluate emotional engagement level of medical students. The predictors related to engagement level were determined by the logistic regression analysis. Furthermore, we constructed a nomogram to predict emotional engagement level of medical students.

**Results:**

A total of 10,576 sample were included in this study. The mean emotional engagement score was 74.61(± 16.21). In the multivariate logistic regression model, we found that males showed higher engagement level compared with females [odds ratio (OR) (95% confidence interval (CI)): 1.263 (1.147, 1.392), *P* < 0.001]. Medical students from the second batches of medical universities had higher engagement level and from “Project 985” universities had lower engagement level compared with 211 project universities [OR (95%CI): 1.376 (1.093, 1.733), *P* = 0.007; OR (95%CI): 0.682 (0.535, 0.868), *P* = 0.002]. Medical students in grade 4 and grade 2 presented lower engagement level compared with in grade 1 [OR (95%CI): 0.860 (0.752, 0.983), *P* = 0.027; OR (95%CI): 0.861 (0.757, 0.980), *P* = 0.023]. Medical students lived in provincial capital cities had higher engagement level compared with in country [OR (95%CI): 1.176 (1.022, 1.354), *P* = 0.024]. Compared with eight-year emotional duration, medical students in other emotional duration (three-year and four-year) had lower engagement level [OR (95%CI): 0.762 (0.628, 0.924), *P* = 0.006]. Medical students’ engagement level increased with increases of grade point average and interest in studying medicine. Medical students learned by converging style showed lower engagement level [OR (95%CI): 0.827 (0.722, 0.946), *P* = 0.006] compared with accommodating style. The model showed good discriminative ability (area under curve = 0.778), calibrating ability and clinical utility.

**Conclusions:**

We identified influencing factors of medical students’ emotional engagement and developed a nomogram to predict medical students’ emotional engagement level, providing reference and convenience for educators to assess and improve emotional engagement level of medical students. It is crucial for educators to pay more attention to emotional engagement of medical students and adopt effective strategies to improve their engagement level.

**Supplementary Information:**

The online version contains supplementary material available at 10.1186/s12909-023-04504-w.

## Introduction

Currently, positive psychology research has become an emerging trend. It focuses on human strengths and optimal functioning rather than on weaknesses and malfunctioning [1]. As a positive mental state, engagement has become hotspots in researches. Emotional engagement refers to a persistent, positive and pervasive emotional and cognitive state related to emotional, scientific research and employment, which is not focused on any particular object, event, individual, or behavior [1, 2]. It is characterized by vigor, dedication and absorption [1]. Emotional engagement is an important indicator of students’ positive psychology in emotional. It can reflect the positive and healthy mental state of students, and is conducive to stimulating students’ positive qualities such as optimism, resilience, sense of meaning and creativity, which effectively promote the maturity and development of students, and lay a solid foundation for them to enter the society [2]. Medical and health service is related to people’s life and health, medical students are the reserve force of the future medical and health service, so medical students shoulder the important mission of improving people’s health. Prior studies have reported that emotional engagement was negatively related to burnout [1, 3], and positively related to well-being [4–6]. Numerous studies have revealed that emotional engagement was positively related to academic achievement, and emotional engagement was a key factor in mediating medical students’ motivation and academic performance [6–8]. Therefore, it is crucial to understand the emotional engagement of medical students and its influencing factors, which will help them achieve better self-worth in their professional fields.

Studies showed that health workers with lower psychological distress, higher job stress, higher levels of perceived social support, psychological flexibility and subjective well-being had higher levels of work engagement [9, 10]. Previous study suggested that emotional engagement of medical student was associated with exercise, sleep, drugs and alcohol use, maintaining relationships, financial stress, thoughts of dropping out and questioning the decision to enter medical school [11]. Besides, learning adaptability and time management disposition were also influencing factors of emotional engagement of medical students [12]. Numerous studies have studied work engagement in health workers, while, rare studies investigated factors affecting emotional engagement based on large samples in medical students. Moreover, current studies only explored some factors influencing emotional engagement among medical students. There were still some influencing factors that have not been further studied, such as educational system, university category and learning style.

Hence, we aimed to perform a cross-sectional study among 10,901 medical students from 11 universities in China to identify influencing factors of medical students’ emotional engagement and construct a nomogram to predict emotional engagement level of medical students.

## Materials and methods

### Study design

The details of study design have been previously published [13]. Briefly, we recruited medical students among 11 universities in China from 20^th^, February 2020 to 31^rd^, March 2020. We randomly selected one or two classes in each grade, and all students in each class were selected to complete the electronic questionnaire.

### Data collection

For each student, the following information was collected, including age, gender, university category, major, ethnicity, whether he/she was an only child, grade, native place, educational system, grade point average (GPA), parental educational level, parental occupation, learning environment of schools, doctor − patient relationship in their hospitals, interests of medicine, Kolb Learning Style and learning engagement level. Among university category, “Project 985” universities refer to the colleges selected into “Project 985” that aims to build universities with world’s advanced level and is research-oriented, and “Project 211” universities refer to the colleges selected into the “Project 211” that aims to construct key universities facing the 21st century. Major included clinical medicine, nursing, phylaxiology, preclinical medicine and stomatology, phylaxiology referred to preventive medicine. Grade referred to the number of study years after entering the university. Native place referred to the place of living. Education system referred to studies duration. Parental occupation including civil servant, company employee, freelance work, individual household, professional/technical and worker/peasant, individual household was the laborer who ran his own business and earned their own living, worker/peasant meant laborer/farmer.

### Assessment tool

The Utrecht Work Engagement Scale (UWES) was widely used internationally and has been translated into multiple languages version [14]. Based on the UWES, Schaufeli et al. developed the UWES-Student (UWES-S) with college students as samples [1]. The Chinese version of UWES-S was used to measure medical students’ emotional engagement level in this study, and it has been confirmed to have good reliability (Cronbach’s alpha = 0.91) and validity [2, 15]. The contents of the scale were shown in Table S1 (English version) and Table S2 (Chinese version). The scale consisted of 3 dimensions (Vigor, Dedication and Absorption) and 17 items. All items were scored on a 7-point Likert scale ranging from 0 (never) to 6 (always). The higher score represented a higher emotional engagement level.

### Statistical analysis

Sample calculation by PASS software identified that a sample size of 1537 participants was needed to achieve a 0.05 significance level. Continuous variables were shown as mean ± standard deviation (SD) and categorical variables as number (percentage). Two independent sample *t*-test and analysis of variance (ANOVA) were analyzed to assess the differences on UWES score related to variables. We divided the samples into low-level and high-level groups according to the median value of UWES score. Firstly, the variables associated with emotional engagement level were identified by the univariate logistic regression analysis. Then, significant variables were integrated into the multivariate logistic regression model, as to identify factors influencing emotional engagement level among medical students. Finally, the nomogram was constructed to predict the probability of high emotional engagement level. Receiver operating characteristic (ROC) curve was performed to assess discriminative ability of the model. Calibration plot was performed to assess calibrating ability. Decision curve analysis (DCA) was performed to assess clinical utility.

In this study, two-sided *P* value < 0.05 was considered as significantly statistical. All statistics analysis processes were performed with R version 3.6.1 (Institute for Statistics and Mathematics, Vienna, Austria).

## Results

### Emotional engagement level of medical students

**Fig. 1:**
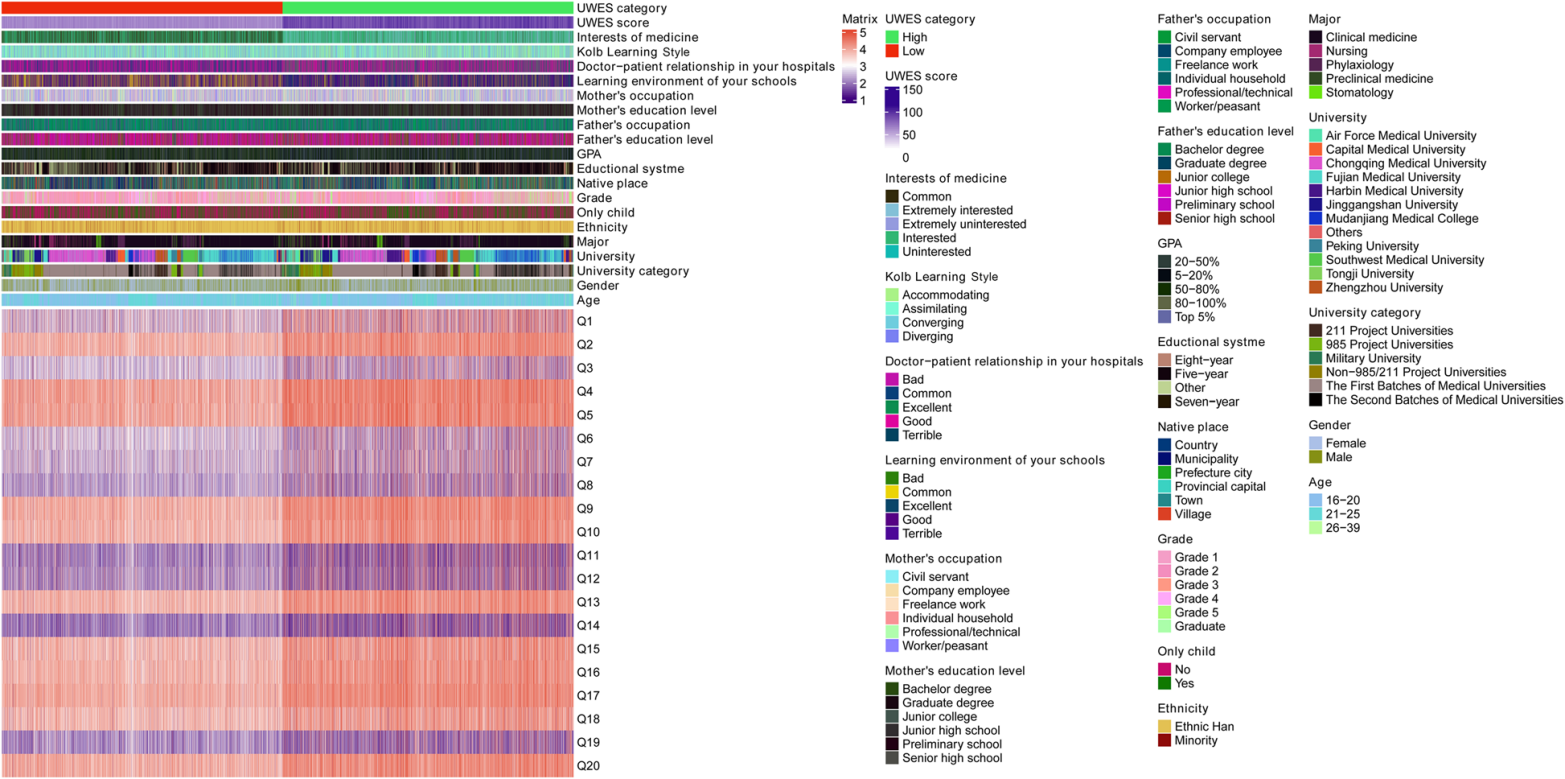
Heatmap of the UWES score. GPA, grade point average; UWES, Utrecht Work Engagement Scale

A total of 10,901 questionnaires were received. After eliminating the questionnaires with outliers and missing values, 10,576 questionnaires were used for the final analysis. The age range of medical students was mainly between 16 and 25 years old (98.79%). A total of 4205 participants were males (39.76%), most medical students were ethnic Han (93.54%) and majoring in clinical medicine (79.15%). A total of 61.20% of medical students were from the First Batches of Medical Universities. The mean UWES score of medical students was 74.61(± 16.21). The analysis results were shown in Table 1 and Fig. 1.

**Table 1:** Emotional engagement level of medical students

**Variables**	**UWES score** **(mean ± SD)**	***P*-value**
**Age**		< 0.001*
16–20 (*n* = 5715)	75.27 ± 16.61	
21–25 (*n* = 4733)	73.69 ± 15.66	
26–39 (*n* = 128)	78.77 ± 15.99	
**Gender**		< 0.001*
Male (*n* = 4205)	76.41 ± 18.27	
Female (*n* = 6371)	73.42 ± 14.57	
**University category**		< 0.001*
Non − 985/211 Project Universities (*n* = 720)	75.99 ± 15.89	
211 Project Universities (*n* = 692)	75.97 ± 16.39	
985 Project Universities (*n* = 853)	70.63 ± 14.89	
Military University (*n* = 526)	77.75 ± 16.19	
The First Batches of Medical Universities (*n* = 6473)	73.60 ± 15.64	
The Second Batches of Medical Universities (*n* = 1312)	79.42 ± 18.36	
**Major**		< 0.001*
Clinical medicine (*n* = 8371)	74.89 ± 16.32	
Nursing (*n* = 567)	70.66 ± 13.69	
Phylaxiology (*n* = 689)	73.49 ± 16.58	
Preclinical medicine (*n* = 652)	74.75 ± 15.64	
Stomatology (*n* = 297)	76.40 ± 16.76	
**Ethnicity**		0.066
Ethnic Han (*n* = 9893)	74.68 ± 16.26	
Minority (*n* = 683)	73.55 ± 15.46	
**Only child**		< 0.001*
No (*n* = 5977)	73.89 ± 15.05	
Yes (*n* = 4599)	75.55 ± 17.56	
**Grade**		< 0.001*
Grade 1 (*n* = 3722)	76.15 ± 16.57	
Grade 2 (*n* = 1986)	74.43 ± 16.73	
Grade 3 (*n* = 1639)	72.72 ± 15.51	
Grade 4 (*n* = 1843)	72.36 ± 15.09	
Grade 5 (*n* = 1254)	76.11 ± 16.33	
Graduate (*n* = 132)	74.31 ± 14.17	
**Native place**		< 0.001*
Village (*n* = 2366)	73.54 ± 14.85	
Town (*n* = 1131)	73.89 ± 15.04	
Prefecture city (*n* = 1974)	76.63 ± 17.07	
Provincial capital (*n* = 1088)	76.23 ± 17.11	
Municipality (*n* = 1484)	72.97 ± 16.13	
Country (*n* = 2533)	74.62 ± 16.68	
**Educational system**		< 0.001*
Five − year (*n* = 7376)	75.51 ± 16.52	
Seven − year (*n* = 280)	73.96 ± 16.37	
Eight − year (*n* = 1281)	73.37 ± 15.91	
Other (*n* = 1639)	71.62 ± 14.47	
**GPA**		< 0.001*
Top 5% (*n* = 758)	80.05 ± 16.94	
5–20% (*n* = 2431)	76.62 ± 15.32	
20–50% (*n* = 3744)	74.91 ± 15.58	
50–80% (*n* = 2640)	72.49 ± 15.76	
80–100% (*n* = 1003)	70.05 ± 18.98	
**Father's education level**		< 0.001*
Preliminary school (*n* = 1769)	72.11 ± 15.08	
Junior high school (*n* = 3721)	74.61 ± 15.50	
Senior high school (*n* = 2514)	75.36 ± 16.85	
Junior college (*n* = 1104)	74.99 ± 16.52	
Graduate degree (*n* = 233)	75.70 ± 19.16	
Bachelor degree (*n* = 1235)	76.10 ± 17.25	
**Father's occupation**		0.001*
Civil servant (*n* = 1032)	75.95 ± 17.07	
Company employee (*n* = 1057)	73.95 ± 17.23	
Freelance work (*n* = 2062)	74.65 ± 15.74	
Individual household (*n* = 1056)	75.86 ± 16.83	
Professional/technical (*n* = 1103)	74.96 ± 16.88	
Worker/peasant (*n* = 4266)	74.02 ± 15.58	
**Mother's education level**		< 0.001*
Preliminary school (*n* = 3126)	73.00 ± 14.985	
Junior high school (*n* = 3241)	74.63 ± 15.59	
Senior high school (*n* = 2159)	75.35 ± 16.92	
Junior college (*n* = 977)	76.64 ± 18.11	
Graduate degree (*n* = 163)	75.83 ± 18.51	
Bachelor degree (*n* = 910)	75.88 ± 17.57	
**Mother's occupation**		< 0.001*
Civil servant (*n* = 599)	75.19 ± 17.251	
Company employee (*n* = 1206)	73.70 ± 17.306	
Freelance work (*n* = 2816)	74.45 ± 15.64	
Individual household (*n* = 770)	75.60 ± 16.37	
Professional/technical (*n* = 1308)	76.52 ± 17.81	
Worker/peasant (*n* = 3877)	74.08 ± 15.43	
**Learning environment of schools**		< 0.001*
Terrible (*n* = 60)	66.42 ± 28.75	
Bad (*n* = 116)	64.22 ± 17.05	
Common (*n* = 2210)	68.06 ± 13.94	
Good (*n* = 5898)	73.16 ± 13.22	
Excellent (*n* = 2292)	85.40 ± 19.20	
**Doctor − patient relationship in your hospitals**		< 0.001*
Terrible (*n* = 45)	65.02 ± 31.60	
Bad (*n* = 117)	66.05 ± 16.24	
Common (*n* = 2753)	68.74 ± 13.56	
Good (*n* = 6009)	73.75 ± 13.49	
Excellent (*n* = 1652)	88.39 ± 20.28	
**Kolb Learning Style**		< 0.001*
Accommodating (*n* = 3572)	76.28 ± 18.00	
Assimilating (*n* = 3119)	73.29 ± 14.22	
Converging (*n* = 1734)	74.06 ± 15.75	
Diverging (*n* = 2151)	74.18 ± 15.91	
**Interests of medicine**		< 0.001*
Extremely uninterested (*n* = 65)	47.40 ± 23.20	
Uninterested (*n* = 161)	54.25 ± 16.38	
Common (*n* = 2599)	64.24 ± 11.02	
Interested (*n* = 5970)	74.55 ± 12.01	
Extremely interested (*n* = 1781)	92.77 ± 17.46	

*UWES* Utrecht Work Engagement Scale, *SD* Standard deviation, *GPA* Grade point average

^*^P < 0.05

### The identification of influencing factors of medical students’ emotional engagement

Firstly, the univariate logistic regression analysis was performed to identify the variables associated with emotional engagement level. We found that 11 variables including gender, university category, major, whether he/she was an only child, grade, native place, educational system, GPA, father occupation, interests of medicine and Kolb Learning Style were associated with emotional engagement of medical students (*P* < 0.05) (Table S3). Then, 11 variables were incorporated into the multivariate logistic regression model. The model suggested that males had higher emotional engagement level [OR (95%CI): 1.263 (1.147, 1.392), *P* < 0.001], compared with females; medical students from “Project 985” universities had lower emotional engagement level and the second batches of medical universities had higher emotional engagement level compared with “Project 211” universities [OR (95%CI): 0.682 (0.535, 0.868), *P* = 0.002; OR (95%CI): 1.376 (1.093, 1.733), *P* = 0.007]; medical students in grade 4 and grade 2 showed lower emotional engagement level compared with in grade1, respectively [OR (95%CI): 0.860 (0.752, 0.983), *P* = 0.027; OR (95%CI): 0.861 (0.757, 0.980), *P* = 0.023]; medical students lived in provincial capital and prefecture city had higher emotional engagement level, respectively [OR (95%CI): 1.176 (1.022, 1.354), *P* = 0.024; OR (95%CI): 1.265 (1.069, 1.498), *P* = 0.006]; the higher the GPA was, the higher the emotional engagement level of medical students were [OR (95%CI):1.267 (1.051, 1.531), *P* = 0.014, for top 5% GPA; OR (95%CI): 1.158 (1.028, 1.304), *P* = 0.016, for 5–20% GPA; OR (95%CI): 0.732 (0.651, 0.822), *P* < 0.001, for 50–80% GPA; OR (95%CI): 0.703 (0.591, 0.836), *P* < 0.001, for 80–100% GPA]; medical students interesting in medicine presented higher emotional engagement level [OR (95%CI): 0.489 (0.261, 0.840), *P* = 0.016, for uninteresting of medicine; OR (95%CI): 6.121 (5.440, 6.899), *P* < 0.001, for interesting of medicine; OR (95%CI): 44.421 (36.653, 54.139), *P* < 0.001, for extremely interesting of medicine]; medical students who learned by accommodating style showed higher engagement level [OR (95%CI): 0.860 (0.768, 0.946), *P* = 0.009, for assimilating; OR (95%CI): 0.827 (0.722, 0.946), *P* = 0.006, for converging; OR (95%CI): 0.857 (0.755, 0.972), *P* = 0.017, for diverging] (Table 2).

**Table 2:** Multivariate logistic regression analysis of emotional engagement level

**Variables**	**Emotional engagement level**	
	**OR (95% CI)**	***P*-value**
**Gender**		
Female	1.00 (reference)	
Male	1.263 [1.147, 1.392]	< 0.001*
**University category**		
211 Project Universities	1.00 (reference)	
985 Project Universities	0.682 [0.535, 0.868]	0.002*
Military University	1.049 [0.797, 1.381]	0.736
Non-985/211 Project Universities	0.971 [0.756, 1.246]	0.815
The First Batches of Medical Universities	0.795 [0.658, 0.961]	0.018*
The Second Batches of Medical Universities	1.376 [1.093, 1.733]	0.007*
**Major**		
Clinical medicine	1.00 (reference)	
Nursing	1.069 [0.838, 1.362]	0.591
Phylaxiology	0.893 [0.741, 1.075]	0.232
Preclinical medicine	1.205 [0.974, 1.492]	0.087
Stomatology	1.321 [0.999, 1.750]	0.051
**Only child**		
No	1.00 (reference)	
Yes	0.954 [0.861, 1.057]	0.369
**Grade**		
Grade 1	1.00 (reference)	
Grade 2	0.861 [0.757, 0.980]	0.023*
Grade 3	0.925 [0.806, 1.060]	0.261
Grade 4	0.860 [0.752, 0.983]	0.027*
Grade 5	1.044 [0.894, 1.220]	0.587
Graduate	0.859 [0.564, 1.312]	0.478
**Native place**		
Country	1.00 (reference)	
Village	1.132 [0.983, 1.304]	0.086
Town	1.095 [0.928, 1.291]	0.281
Prefecture city	1.176 [1.022, 1.354]	0.024*
Provincial capital	1.265 [1.069, 1.498]	0.006*
Municipality	1.031 [0.882, 1.205]	0.700
**Educational system**		
Eight year	1.00 (reference)	
Seven year	0.887 [0.636, 1.235]	0.477
Five year	1.075 [0.928, 1.246]	0.334
Other	0.762 [0.628, 0.924]	0.006*
**GPA**		
20 − 50%	1.00 (reference)	
top 5%	1.267 [1.051, 1.531]	0.014*
5 − 20%	1.158 [1.028, 1.304]	0.016*
50 − 80%	0.732 [0.651, 0.822]	< 0.001*
80 − 100%	0.703 [0.591, 0.836]	< 0.001*
**Father’s occupation**		
Civil servant	1.00 (reference)	
Company employee	0.852 [0.695, 1.045]	0.124
Freelance work	0.863 [0.719, 1.036]	0.115
Individual household	0.906 [0.737, 1.114]	0.350
Professional/technical	0.888 [0.725, 1.088]	0.253
Worker/peasant	0.838 [0.702, 1.000]	0.050
**Interests of medicine**		
Common	1.00 (reference)	
Extremely uninterested	0.630 [0.259, 1.308]	0.256
Uninterested	0.489 [0.261, 0.840]	0.016*
Interested	6.121 [5.440, 6.899]	< 0.001*
Extremely interested	44.421 [36.653, 54.139]	< 0.001*
**Kolb Learning Style**		
Accommodating	1.00 (reference)	
Assimilating	0.860 [0.768, 0.964]	0.009*
Converging	0.827 [0.722, 0.946]	0.006*
Diverging	0.857 [0.755, 0.972]	0.017*

*OR* Odds ratio, *CI* Confidence interval, *GPA* Grade point average

^*^P < 0.05

### The nomogram and validation

**Fig. 2:**
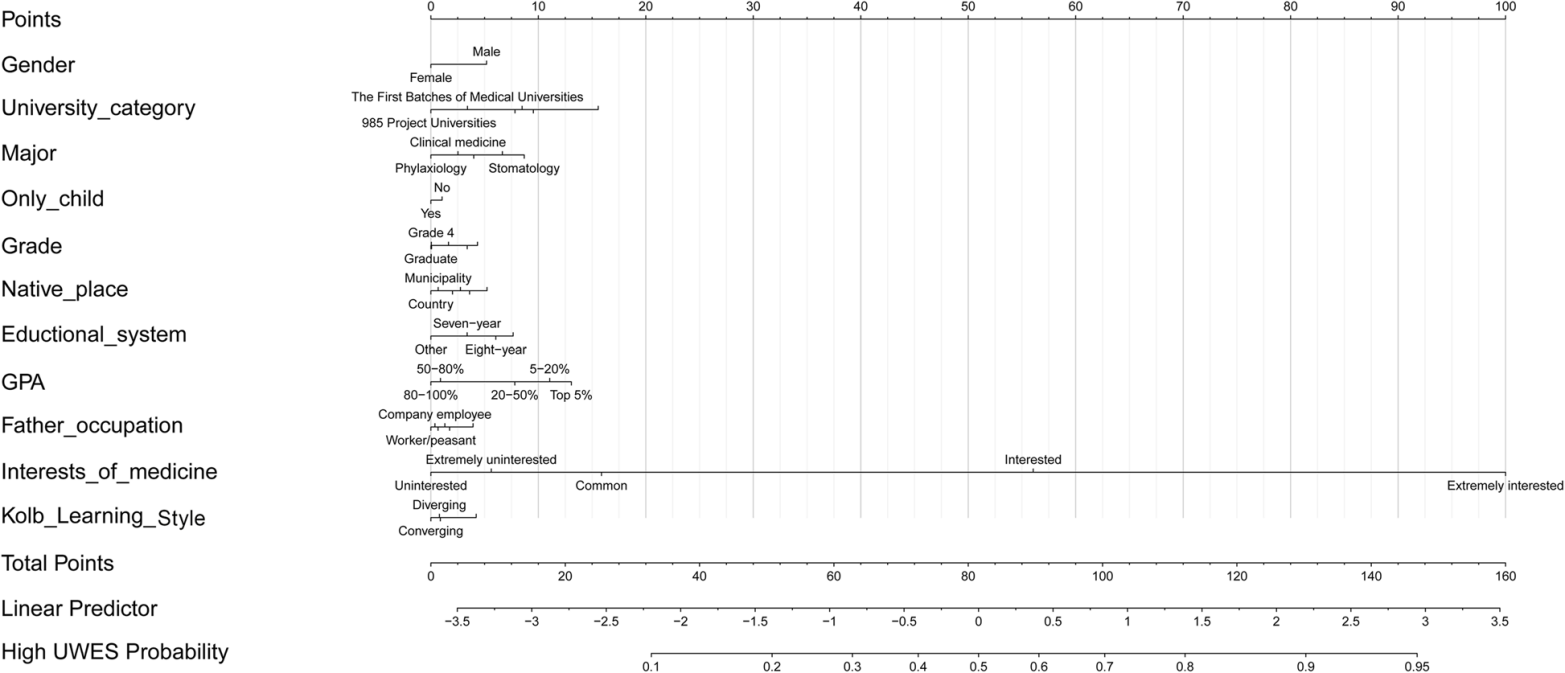
Nomogram of predicting emotional engagement level of medical students. GPA, grade point average; UWES, Utrecht Work Engagement Scale

**Fig. 3:**
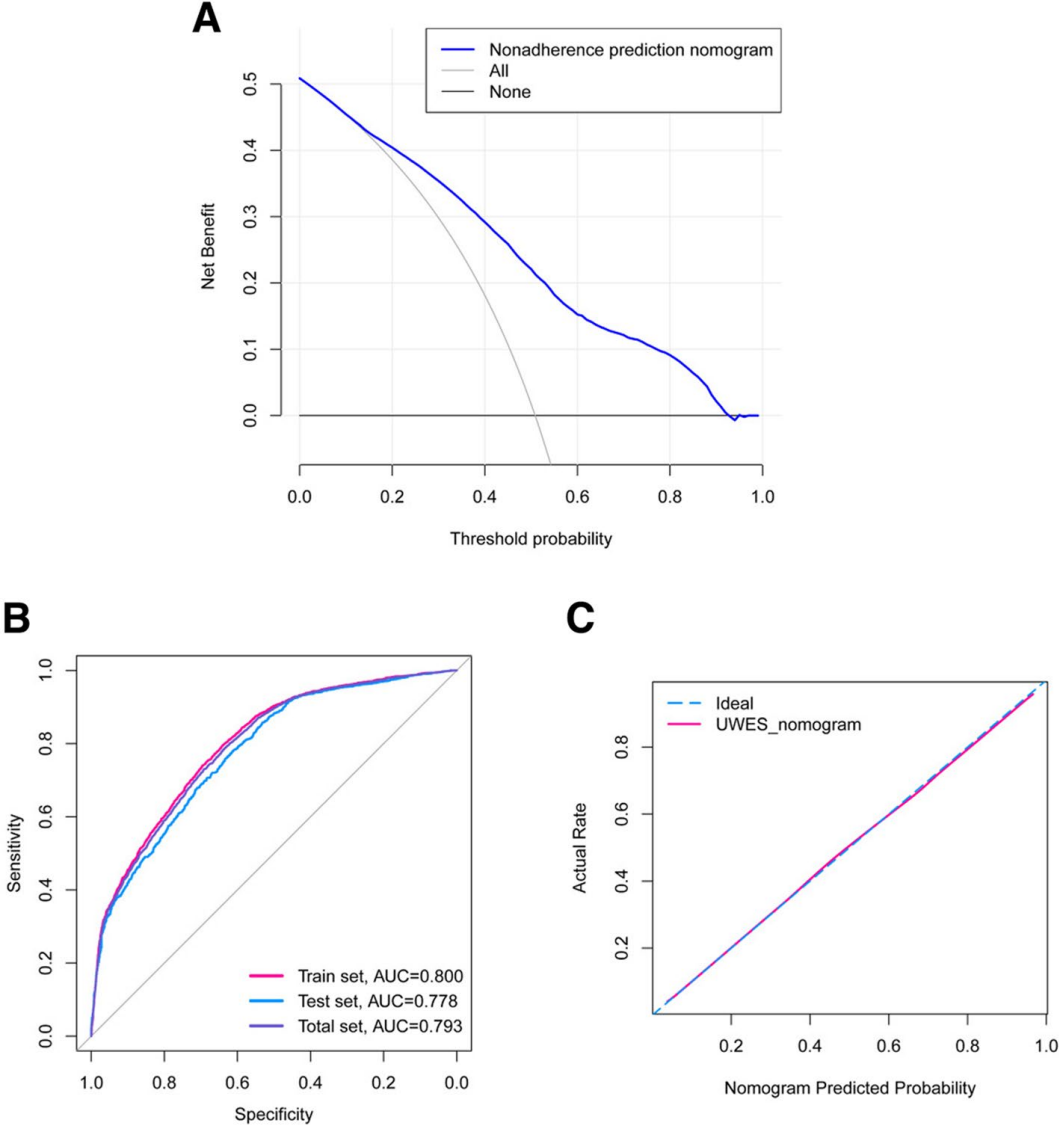
The nomogram validation. **A** DCA of the nomogram showed the model had good clinical utility. **B** AUC of ROC indicated that the model had good discriminative ability (Total set AUC = 0.793, Train set AUC = 0.800, Test set AUC = 0.778). **C** The calibration curve of the nomogram presented the model had good predictive consistency. DCA, decision curve analysis; UWES, Utrecht Work Engagement Scale; ROC, receiver operating characteristic; AUC, area under the curve

We established the nomogram based on the variables determined by the univariate logistic regression analysis (Fig. 2). DCA indicated that the model had good clinical utility (Fig. 3A). The ROC curve showed that the model had sufficient discriminative ability (area under curve (AUC) of train set = 0.800, AUC of test set = 0.778) (Fig. 3B). The calibration curve showed good consistency between the predicted emotional engagement level of the model and the actual emotional engagement level (Fig. 3C).

## Discussion

Academic burden of medical students is relatively large. Engagement in learning emotional may be more conducive to achieve better academic performance for them and avoid burnout and anxiety on study and life [11, 16]. Therefore, determining influencing factors of leaning emotional engagement can help medical schools take measures to enhance emotional engagement level of medical students and cultivate more outstanding medical talents.

We carried out a multi-center cross-sectional study to investigate influencing factors on emotional engagement of medical students. The results indicated that gender, university category, grade, native place, educational system, GPA, interests of medicine and Kolb Learning Style were predictors of emotional engagement. Furthermore, we constructed the nomogram to predict the emotional engagement level of medical students, and the nomogram model showed good discriminative and calibrating ability and clinical utility.

### Gender and emotional engagement of medical students

There was gender difference in emotional engagement level, and males had higher emotional engagement level compared with females. Medical students’ intrinsic motivation in medicine positively correlated with emotional engagement level, and the male showed higher intrinsic motivation than females, such as having a strong interest in medicine and being confident I can succeed in the medicine filed [8, 17]. In addition, males perceived higher emotional and emotional process support from teachers than females, and the support from teachers had a beneficial impact on emotional engagement of students [18, 19]. However, some studies suggested that females showed higher engagement level in emotional than males [20, 21]. But, studies on gender difference of emotional engagement level in medical students were limited. Thus, more large-sample studies should be conducted to investigate the influence of gender on emotional engagement level of medical students.

### Native place and emotional engagement of medical students

Compared with medical students from country, students from provincial capital and prefecture cities presented higher emotional engagement level. That might be related to some social and psychological factors, such as the local education level, parents’ education level, and family environment. Medical students from provincial capital or prefecture cities might receive more social support, which was conductive to improve emotional engagement level [22]. Moreover, prior study also reported medical students from cities showed significantly higher learning motivation that was positively associated with emotional engagement [23, 24].

### University-related and student-related factors (university category, grade, educational system, GPA, interests of medicine and learning style) and emotional engagement of medical students

In this study, compared with medical students from “Project 211” university, medical students from “Project 985” universities had lower emotional engagement level, followed by the first batches of medical universities, while medical students from the second batches of medical university had higher emotional engagement level. The impact of university category on emotional engagement might be related to the universities’ training goals for students, learning environment of universities and the degree of caring about the physical and mental health of students [25, 26]. Medical students from “Project 985” university might suffer from more academic stress, and stress caught directly affect engagement in learning emotional [27]. At present, studies investigating the impact of university category on the emotional engagement were limited, therefore, it might be necessary to conduct more research on the impact of university category on emotional engagement and related reasons.

Before 2015, China’s medical education system were divided by learning duration, including five-year medicine program leading to a Bachelor’s of medicine degree, seven-year medicine program leading to a Master’s of medicine degree and eight-year medicine program leading to a Doctor of medicine degree. Moreover, to improve medical education quality, all seven-year degree programs were changed into ‘5 + 3’ master degree programs in 2015 [28]. In this study, students in grade 2 and grade 4 had lower emotional engagement level compared with in grade 1, which was consistent with previous study [29]. Most medical students were education periods of five year in this study, they caught face internships, future academic thinking, and accumulated academic and mental pressures in the fourth year [30], which might make them more prone to burnout. Besides, burnout was an important predictor of emotional engagement [31, 32]. Prior study also revealed that medical students at higher-grade experienced increased burnout risk and decreased emotional engagement level [3]. Study also reported that medical students in higher grade experienced higher depression and stress levels compared with in grade 1, which would affect students’ emotional engagement [33]. Depression and stress had negative effect on emotional engagement [34]. Furthermore, this phenomenon might also indicate that medical students have started to experience a decline in emotional engagement level from the early stage. Thus, the medical schools should take measures from the early stage to prevent the decline of medical students’ emotional engagement level. Additionally, this study revealed that eight-year medical students reported higher emotional engagement level in this study. This indicated that the training mode of eight-year clinical medical students might have a positive influence on emotional engagement level of medical students. The educational goals of the eight-year medical program are to cultivate medical talents with a solid foundation in medical theory, strong self-learning ability, practical ability, clinical ability, scientific research ability, communication ability as well as good innovation spirit and comprehensive quality. Due to the longer learning time of the eight-year program, medical students caught achieve better coherence in learning contents and more comprehensive and in-depth understanding on research project. That might be beneficial for cultivating medical students’ emotional engagement. There was a lack of evidence about the relationship between educational system and emotional engagement, so further research was needed to explore their relationship.

The results reported that students with higher GPA and great interest in medicine had higher emotional engagement level, which were consistent with previous studies [35, 36].These students tended to have greater learning motivation and more effective learning strategies [23]. That caught drive them to have a better engagement in emotional.

Medical students with accommodating learning style had higher emotional engagement level compared with other learning styles. kolb conducted an analysis of the learning cycle, and believed the learning cycle was composed of four interconnected links, namely concrete experience (CE), reflective observation (RO), abstract conceptualization (AC) and active experimentation (AE). CE where the student learned through involvement in experience; RO where the student learned through watching and making sense of experience; AC where the student learned by connecting past experience and ideas, and forming an opinion on what that means to them, and considering what could have been done to enhance the outcome; AE where the student directed future practice by using what they have learned. These four links would have different combinations of preferences in each body, thus showing different learning styles: accommodating (CE and AE), assimilating (AC and RO), converging (AC and AE) and diverging (CE and RO) [37, 38]. Individuals with accommodating learning style learned through hands-on experiences and previous attained experience and were willing to devote themselves to new or challenging jobs [39]. Hands-on was helpful to strengthen the connection between teaching and clinic and an important means to cultivate medical students’ clinical ability and skills. Hands-on skills were an important basis for medical students in hospital. Hands-on caught strengthen sensory/perceptual experiences and help to recall more information [40]. Therefore, universities should attach importance to and enhance the experience of clinical practice of medical students in order to increase students’ engagement in emotional.

This was a multiple-center large sample to identify influencing factors of medical students’ emotional engagement in China. Thus, the results were more representative. Furthermore, we constructed the nomogram and it provided a convenient way to evaluate the emotional engagement level of medical students. However, there were still some limitations in this study that should be taken into account in interpreting the results. Firstly, this study used a cross-sectional study design, so it was difficult to explain the causality between influencing factors and emotional engagement. Secondly, influence of cohort effects on results could not be completely dismissed. Thus, prospective studies were recommended to solve the limitation. Thirdly, we used self-reporting questionnaires to gather information, students might exaggerate or reduce self-report contents. Fourthly, we did not comprehensively collect the characteristic information of the participants, which might lead to the existence of residual confounding. Fifthly, the results were able to be generalized to students in other healthcare disciplines. Finally, factors identified by the univariate logistic regression analysis were used to construct the nomogram in this study, which might cause overfitting.

## Conclusion

In this study, we identified influencing factors of medical students’ emotional engagement, including gender, university category, grade, native place, educational system, GPA, interests of medicine and Kolb Learning Style. Besides, we constructed the nomogram to predict the emotional engagement level of medical students. This study provides help to intervene and improve the emotional engagement of medical students. More studies exploring other influencing factors of emotional engagement of medical students are warranted.

### Supplementary Information

**Additional file 1: Table S1.** Utrecht work engagement scale - student version (English version). **Table S2.** Utrecht work engagement scale - student version (English version). **Table S3.** Univariate logistic regression analysis of learning engagement level.

## Data Availability

The datasets generated and/or analyzed during the current study are available in the supplementary material.
