# Supplementary material for: A multi-center cross-sectional study on identification of influencing factors of medical students’ emotional engagement in China
Source: BMC Med Educ. 2023 Nov 7;23:838. doi: 10.1186/s12909-023-04504-w (PMC10631166; doi:10.1186/s12909-023-04504-w)
Supplement: Supplementary file 1 — Additional file 1: Table S1. Utrecht work engagement scale - student version (English version). Table S2. Utrecht work engagement scale - student version (English version). Table S3. Univariate logistic regression analysis of learning engagement level. [file 12909_2023_4504_MOESM1_ESM.docx]

| Scale items |
| --- |
| **Vigor** |
| 1. When I get up in the morning, I feel like going to class. |
| 2. When I'm doing my work as a student, I feel bursting with energy. |
| 3. As far as my studies are concerned I always persevere, even when things do not go well. |
| 4. I can continue studying for very long periods at a time. |
| 5. I am very resilient, mentally, as far as my studies are concerned. |
| 6. I feel strong and vigorous when I'm studying or going to class.  **Dedication** |
| 7. To me, my studies are challenging. |
| 8. My study inspires me. |
| 9. I am enthusiastic about my studies. |
| 10. I am proud of my studies. |
| 11. I find my studies full of meaning and purpose  **Absorption** |
| 12. When I am studying, I forget everything else around me. |
| 13. Time flies when I am studying. |
| 14. I get carried away when I am studying. |
| 15. It is difficult to detach myself from my studies. |
| 16. I am immersed in my studies. |
| 17. I feel happy when I am studying intensely. |

**Table S1 Utrecht work engagement scale - student version (English version)**

**Table S2 Utrecht work engagement scale - student version (English version)**

The following 17 sentences are statements about your experience in studying. Please read it carefully and determine if you have ever felt this way while studying. Check "0" if you have never felt this way. If you have felt this way, tick the number (from 1 to 6) that best describes how often you feel.

| Questions | Never | Hardly ever/less | Rarely/once a month or less | Sometimes  /several times a month | Often  /once a week | Very often  /several times a week | Always  /every day |
| --- | --- | --- | --- | --- | --- | --- | --- |
| 1. I feel myself bursting with energy in learning. | 0 | 1 | 2 | 3 | 4 | 5 | 6 |
| 2. I feel that the learning I am engaged in is purposeful and meaningful. | 0 | 1 | 2 | 3 | 4 | 5 | 6 |
| 3. Time always flies when I'm studying. | 0 | 1 | 2 | 3 | 4 | 5 | 6 |
| 4. When I study, I feel strong and energetic. | 0 | 1 | 2 | 3 | 4 | 5 | 6 |
| 5. I have a passion for learning. | 0 | 1 | 2 | 3 | 4 | 5 | 6 |
| 6. I forget everything around me when I study. | 0 | 1 | 2 | 3 | 4 | 5 | 6 |
| 7. Learning inspires me. | 0 | 1 | 2 | 3 | 4 | 5 | 6 |
| 8. As soon as I get up in the morning, I want to study. | 0 | 1 | 2 | 3 | 4 | 5 | 6 |
| 9. I will feel happy when I am nervous about studying. | 0 | 1 | 2 | 3 | 4 | 5 | 6 |
| 10. I'm proud of the learning I've done. | 0 | 1 | 2 | 3 | 4 | 5 | 6 |
| 11. I am immersed in my study. | 0 | 1 | 2 | 3 | 4 | 5 | 6 |
| 12. I can study continuously for a long time. | 0 | 1 | 2 | 3 | 4 | 5 | 6 |
| 13. For me, my study is challenging. | 0 | 1 | 2 | 3 | 4 | 5 | 6 |
| 14. I can reach a state of selflessness when I study. | 0 | 1 | 2 | 3 | 4 | 5 | 6 |
| 15. When studying, even if I feel mentally tired, I can recover quickly. | 0 | 1 | 2 | 3 | 4 | 5 | 6 |
| 16. I feel that I can't do without studying. | 0 | 1 | 2 | 3 | 4 | 5 | 6 |
| 17. Even if the study is not smooth, I am not discouraged, can persevere. | 0 | 1 | 2 | 3 | 4 | 5 | 6 |

**Table S3 Univariate logistic regression analysis of learning engagement level**

| Variables | *P*-value |
| --- | --- |
| Age | 0.920 |
| Gender | <0.001* |
| University category | <0.001* |
| Major | <0.001* |
| Ethnicity | 0.951 |
| Only child | 0.013* |
| Grade | <0.001* |
| Native place | 0.047* |
| Educational system | <0.001* |
| GPA | <0.001* |
| Father education level | 0.202 |
| Father occupation | 0.002* |
| Mother education level | 0.994 |
| Mother occupation | 0.739 |
| Learning environment of your schools | 0.788 |
| Doctor patient relationship in your hospitals | 0.505 |
| Interests of medicine | <0.001* |
| Kolb Learning Experience | <0.001* |

GPA, grade point average. * *P* < 0.05.
